# Antagonistic roles of cGAS/STING signaling in colorectal cancer chemotherapy

**DOI:** 10.3389/fonc.2024.1441935

**Published:** 2024-10-14

**Authors:** Beiyuan Liang, Xuanxuan Xing, Hayden Storts, Zhen Ye, Hazel Claybon, Ryan Austin, Rachel Ding, Bei Liu, Haitao Wen, Wayne O. Miles, Richard Fishel, Jing J. Wang

**Affiliations:** ^1^ Department of Cancer Biology and Genetics, The Ohio State University, Columbus, OH, United States; ^2^ Division of Hematology, Department of Internal Medicine, The Ohio State University, Columbus, OH, United States; ^3^ Pelotonia Institute for Immuno-Oncology, James Comprehensive Cancer Center, Wexner Medical Center, The Ohio State University, Columbus, OH, United States; ^4^ Department of Microbial Infection and Immunity, The Ohio State University, Columbus, OH, United States

**Keywords:** colorectal cancer, chemoresistance, PD-L1, STING, cGAS, 5-FU/oxaliplatin, anti-tumor immunity, immune checkpoint blockade

## Abstract

FOLFOX, composed of 5-FU, oxaliplatin and leucovorin, is a first line chemotherapy regimen for colorectal cancer (CRC) treatment. In this study, we show that 5-FU and oxaliplatin induce DNA damage and activate cGAS/STING signaling leading to enhanced expression of interferon (IFN) β, IFN-stimulated genes and inflammatory cytokines in mouse and human colon cancer cells as well as increased intratumoral CD8^+^ T cells in mice. Crucially, 5-FU and oxaliplatin increase PD-L1 expression at the mRNA and protein levels, which has been shown to inhibit CD8^+^ T cell function. Depletion of cGAS, STING, IRF3, or IFNα/β receptor 1 (IFNAR1) abolishes this increase, indicating that 5-FU/oxaliplatin mediated upregulation of PD-L1 expression is dependent on tumor cell intrinsic cGAS/STING signaling. These results imply opposing roles for FOLFOX during cancer treatment. On one hand, 5-FU and oxaliplatin activate the innate immune response to facilitate anti-tumor immunity, and conversely upregulate PD-L1 expression to evade immune surveillance. Analysis of TCGA colon cancer dataset shows a positive correlation between expression of PD-L1 and components of the cGAS/STING pathway, supporting a role for cGAS/STING signaling in upregulating PD-L1 expression in colon cancer patients. Tumor studies in syngeneic immune competent mice demonstrate that the combination of 5-FU/oxaliplatin and anti-PD-1 significantly reduced tumor growth of colon cancer cells compared to 5-FU/oxaliplatin treatment alone. Taken together, our studies have identified a unique pathway leading to chemoresistance and provide a rationale to combine FOLFOX with anti-PD-1/PD-L1 as an effective CRC treatment.

## Introduction

Colorectal cancer (CRC) is the third most common cancer and second leading cause of cancer death in the US. FOLFOX, composed of 5-FU, oxaliplatin and leucovorin, is a first line chemotherapy regimen for CRC treatment. Unfortunately, therapeutic resistance remains a significant problem, especially for metastatic CRC. Immunotherapies have shown efficacy in a variety of cancers including CRC (1, 2). Inhibitors targeting PD-1/PD-L1 immune checkpoint have resulted in improved survival of patients presenting with a variety of cancers including CRC. Unfortunately only 20-40% of patients show beneficial effect (3–5). The resistance mechanism(s) of chemotherapy and immunotherapies are largely unknown and underline the need to develop new therapeutic strategies.

PD-1 is an inhibitory receptor expressed by antigen-stimulated T cells. After binding to its ligands, PD-L1/PD-L2, PD-1 mediates downstream signaling that inhibits T cell activation and proliferation, and blocks the anti-tumor immune response (6–8). PD-L1 has been implied to be the dominant inhibitory ligand of PD-1. Cancer cells instigate immune tolerance by inducing expression of PD-L1 (8, 9). Disruption of the interaction between PD-1 and PD-L1 by PD-1 or PD-L1 antibodies enhances T cell-mediated anti-tumor activity and induces durable tumor remissions (10). Expression of PD-L1 on tumor cells and/or in the tumor microenvironment has been associated with clinical response to anti-PD-1/PD-L1 therapies (11). PD-L1 expression has been shown to be regulated by several mechanisms including genomic alterations, transcriptional regulation, post-transcriptional and post-translational modifications (12). Given the importance of PD-L1 expression in tumor control and predicting treatment response, it is crucial to understand the plethora of mechanisms that may regulate PD-L1 expression.

Cyclic GMP-AMP (cGAMP) synthase (cGAS) is a cytosolic DNA sensor that produces the second messenger cGAMP, which binds to and activates the adaptor stimulator of interferon genes (STING). STING is an endoplasmic reticulum (ER) protein that subsequently activates the Tank-binding kinase-1 (TBK1) and IκB kinase (IKK), leading to the activation of transcription factors IRF3 and NF-κB, respectively. Together and/or separately, IRF3 and NF-κB induce the expression of type I interferons (IFNα and IFNβ) that bind to interferon receptor type I (IFNRI). IFNRI is composed of two subunits, IFNAR1 and IFNAR2, that signal through the Janus kinase (JAK)/STAT pathway to regulate the expression of IFN-stimulated genes (ISGs), cytokines, and chemokines that initiate immune responses (13, 14). Numerous studies have demonstrated that DNA damage and genomic instability activate the cGAS/STING pathway (15–17). cGAS/STING activation is essential for efficient cancer therapy including radiation, chemotherapy and anti-PD-1/PD-L1 therapies (18, 19). One of the mechanisms by which cGAS/STING enhances anti-PD-1/PD-L1 efficacy is by increasing tumor infiltrating lymphocytes (TILs) (19). The absence of TILs in CRC has been linked to therapeutic resistance (20–22). Moreover, the cGAS/STING signaling pathway is frequently suppressed or inactivated in a variety of cancers, including CRC (18, 23). Conversely, drugs or genes that activate cGAS/STING signaling may synergize with anti-PD-1/PD-L1 therapies and show increased efficacy (24–26).

5-FU and oxaliplatin are principal chemotherapeutic drugs in the FOLFOX chemotherapy. 5-FU mainly functions as a thymidylate synthase inhibitor, blocking synthesis of the pyrimidine thymidylate (dTMP) that is essential for DNA replication (27). Oxaliplatin is a DNA intra-strand crosslinker that disrupts replication and transcription (28). FOLFOX has been shown to ameliorate CD8^+^ T lymphocyte exhaustion, induce tumor infiltration of activated PD-1^+^ CD8^+^ T cells, and suppress myeloid-derived suppressor cells (MDSCs), all of which contributed to enhanced anti-PD-1 efficacy in mouse models (29–31). The molecular mechanism(s) that contribute to the FOLFOX immune regulatory response remain largely unknown.

Here we show that 5-FU and oxaliplatin induce DNA damage and activate cGAS/STING signaling leading to elevated expression of IFNβ, ISG15 and CXCL10 in mouse and human colon cancer cells as well as increased intratumoral CD8^+^ T cells in mice. Importantly, 5-FU and oxaliplatin increase PD-L1 expression at the mRNA and protein levels, which we find is at least partially dependent on cGAS/STING signaling. Moreover, analysis of TCGA colon cancer dataset supports a role for cGAS/STING signaling in upregulating PD-L1 expression. Taken together, these results indicate that, on one hand, 5-FU and oxaliplatin activate innate immune response to facilitate anti-tumor immunity and, on the other hand, they upregulate PD-L1 expression to suppress immune surveillance. To determine whether combining 5-FU/oxaliplatin with anti-PD-1/PD-L1 might enhance therapy efficacy, we perform tumor studies in syngeneic immune competent mice. We found that the combination of 5-FU/oxaliplatin and anti-PD-1 significantly reduced tumor growth of murine colon cancer cells compared to 5-FU/oxaliplatin treatment alone. Understanding the mechanisms of these unique chemoresistance pathways provides a rationale for combining FOLFOX with anti-PD-1/PD-L1 for efficient CRC treatment.

## Materials and methods

### Colon cancer cells

Mouse colon cancer cell lines, MC38 (RRID: CVCL_B288) and CT26 (RRID: CVCL_7256), and human colon carcinoma cell line, HT29 (RRID: CVCL_A8EZ), were purchased from ATCC. All cell lines were authenticated by STR analyses at Ohio State University Genomics Shared Resources. STR profiles were cross-checked with the ATCC database. MC38, CT26 and HT29 cell lines displayed ≥ 80% match, which is considered valid (32). Cells were tested for mycoplasma every three months with MycoAlert^®^ PLUS Mycoplasma Detection Kit (Lonza Cat# LT07-418).

Cells were maintained at 37°C in a humidified incubator with 5% CO2. MC38 and CT26 cells were cultured in DMEM supplemented with 10% FBS while HT29 cells in McCoy’s 5A medium (Cytiva, Cat# SH30200.01) with 10% FBS. Cells were passaged 2-5 times between thawing and use in the described experiments.

### Antibodies and reagents

The information of antibodies used in this study is as following: anti-STING (Cell Signaling Technology Cat# 13647, RRID: AB_2732796), anti-phospho-STING (mouse: Cell Signaling Technology Cat# 72971, RRID: AB_2799831, human: Cell Signaling Technology Cat# 19781, RRID: AB_2737062), anti-TBK1 (Cell Signaling Technology Cat# 3013, RRID: AB_2199749), anti-phospho-TBK1 (Cell Signaling Technology Cat #5483, RRID: AB_10693472), anti-ISG15 (Santa Cruz Biotechnology Cat# sc-166755, RRID: AB_2126308), anti-STAT1 (Cell Signaling Technology Cat# 9172, RRID: AB_2198300), anti-phospho-STAT1 (Cell Signaling Technology Cat# 9167, RRID: AB_561284), anti-cGAS (Cell Signaling Technology Cat# 31659, RRID: AB_2799008), anti-PD-L1 (mouse: Abcam Cat# ab213480, RRID: AB_2773715; human: Cell Signaling Technology Cat# 13684, RRID: AB_2687655), anti-p65 (Cell Signaling Technology Cat# 8242, RRID: AB_10859369), anti-IRF3 (Cell Signaling Technology Cat# 4302, RRID: AB_331982) and anti-Actin (Santa Cruz Biotechnology Cat# sc-47778 HRP, RRID: AB_2714189). 5-FU and oxaliplatin were purchased from Sigma-Aldrich (Cat# F6627 and O9512).

### Western blot analysis and real time Q-PCR

Whole cell lysates were prepared in RIPA buffer (MilliporeSigma, Cat# 20-188) supplemented with a protease inhibitor cocktail (Thermo Fisher Scientific, Cat# 78430). Equivalent amounts of protein were separated by SDS–PAGE and transferred to a Nitrocellulose membrane (Bio-Rad Cat# 1620115). Proteins were detected using an enhanced chemiluminescence system (LI-COR Biosciences).

Total RNA was isolated, and reverse transcribed to cDNA. Q-PCR analysis was performed using PowerUp™ SYBR™ Green Master Mix (Thermo Fisher Scientific Cat# A25777). The primer sequences for mouse IFNβ, CXCL10 and PD-L1 are ATGAGTGGTGGTTGCAGGC-F, TGACCTTTCAAATGCAGTAGATTCA-R; AGTAACTGCCGAAGCAAGAA-F, GCACCTCCACATAGCTTACA-R and CGCCTGCAGATAGTTCCCAA-F, ATCGTGACGTTGCTGCCATA-R respectively. The primer sequences for human IFNβ, CXCL10 and PD-L1 are ATGACCAACAAGTGTCTCCTCC-F, GGAATCCAAGCAAGTTGTAGCTC-R; AGCAGAGGAACCTCCAGTCT-F, ATGCAGGTACAGCGTACAGT-R and ACAATTAGACCTGGCTGCAC-F, TCAGTGCTACACCAAGGCAT-R respectively. Actin was used as an endogenous control.

### CRISPR/Cas9 knockout

The Alt-R CRISPR-Cas9 System (Integrated DNA Technologies) was used, in which guide RNA contains crRNA with specific DNA target sequence and tracrRNA labeled with ATTO™ 550 (ATTO-TEC). Two crRNAs targeting mouse *Cgas* (ACGGAGAAGCCACGTGCCCC and AAACGGGAGTCGGAGTTCAA), *Sting1* (CACCTAGCCTCGCACGAACT and GTGCCCAGGGCGTCTCCTTG), *Ifnar1* (TCAGTTACACCATACGAATC and GCTTCTAAACGTACTTCTGG) or *Rela* (TGTTCGATGATCTCCACATA and ATCGAACAGCCGAAGCAACG) and human *STING1* (GCTGGGACTGCTGTTAAACG and CATATTACATCGGATATCTG) or *IRF3* (GAGGTGACAGCCTTCTACCG) were synthesized. Guide RNAs complexed with Cas9 protein were transfected into MC38 cells by electroporation. ATTO™ 550-positive cells were sorted into pools by flow cytometry the next day. One week later, cells were harvested and cGAS/STING expression and downstream signaling was determined by western blot analysis to determine knockout efficiency.

### *In vivo* tumor model

Mouse experiments involving animals were approved by the Ohio State University Institutional Animal Care and Use Committee (IACUC) and IACUC regulations were followed. Exponentially growing MC38 cells (0.5 × 10^6^) were inoculated subcutaneously into the flank of 6-7-week-old female C57BL/6J (RRID: IMSR_JAX:000664) mice on one side. Mice was randomly divided into four groups and treated with 1) saline and control IgG, 2) 5-FU/oxaliplatin, 3) anti-PD-1 antibody, or 4) 5-FU/oxaliplatin plus anti-PD-1 antibody. On days 7, 10 and 13, mice were treated with either saline or 5-FU (25mg/kg) plus oxaliplatin (2.5mg/kg) by intraperitoneal (IP) injection. On days 8, 11 and 14, mice were treated with 200 μg of either control IgG (ichorbio Cat# ICH2244, RRID: AB_2921379) or anti-PD-1 antibody (ichorbio Cat# ICH1091, RRID: AB_2921476) by IP injection. Tumors were monitored and measured every other day. Tumor volumes (V) were calculated by the formula V = W^2^ × L × 0.5, where W represents the largest tumor diameter and L represents the next largest tumor diameter.

### Immunohistochemistry staining

Formalin-fixed paraffin-embedded blocks of tumors were cut into 4-micron thick tissue sections. After antigen retrieval using Antigen Unmasking Solution (Vector Laboratories Cat# H-3300, RRID: AB_2336226), tissue slides were blocked with 10% Goat Serum (Vector Laboratories Cat# S-1000, RRID: AB_2336615) diluted in 1X PBS, followed by incubation with an anti-CD8 antibody (Cell Signaling Technology Cat# 98941, RRID: AB_2756376) or an anti-STAT1 antibody (Cell Signaling Technology Cat# 9172, RRID: AB_2198300) overnight at 4°C. The slides were developed with ImmPACT**^®^** DAB Substrate Kit (Vector Laboratories Cat# SK-4105, RRID: AB_2336520) and counterstained with hematoxylin (Leica biosystems Cat# 3801570). Five fields/slide were randomly selected for quantification by ImageJ (RRID: SCR_003070). Percentage of CD8^+^ or nuclear STAT1^+^ cells was calculated as [(CD8^+^ or nuclear STAT1^+^ cell number/total cell number in the field) x 100%].

### Bioinformatics analysis of clinical data

RNA-seq data for TCGA-COAD samples (n =471) was accessed through the Genomic Data Commons Data Portal (GDC Data Portal) (RRID: SCR_014514) (https://portal.gdc.cancer.gov/) (33). Correlation of PD-L1 with other genes was calculated with Spearman’s correlation. Analysis was conducted using R and RStudio.

### Statistical analysis

Experiments were performed a minimum of two times independently and represented by Mean ± SD. Statistics analysis was performed in GraphPad Prism 5 (RRID: SCR_002798). Student’s t-test or ANOVA were used to analyze the differences among groups. Statistically significant differences are indicated as follows: *P<0.05, **P<0.01, ***P<0.001, ****P<0.0001.

## Results

### 5-FU and oxaliplatin activate cGAS/STING and increase IFNβ expression in colon cancer cells

**Figure 1:**
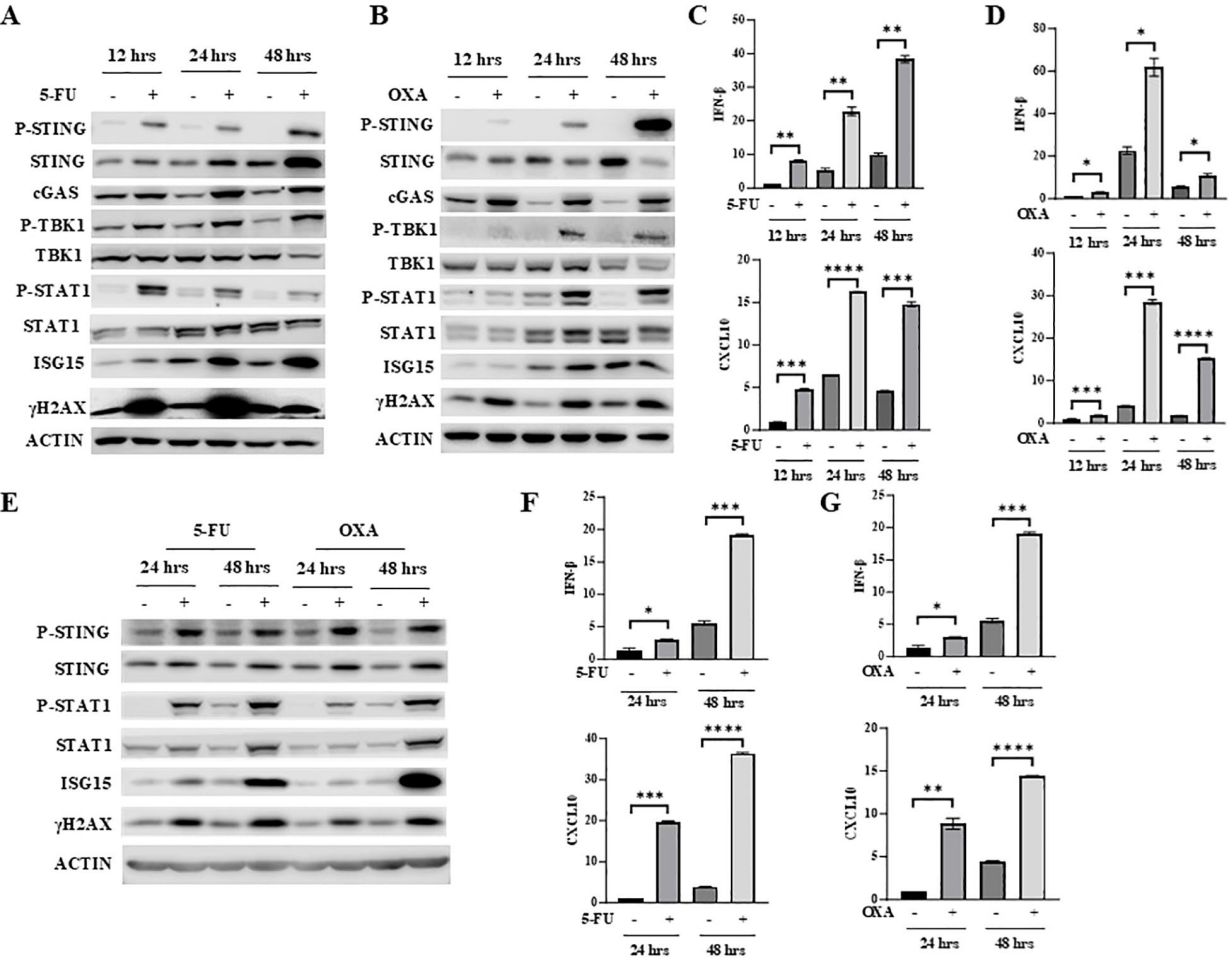
5-FU and oxaliplatin activate cGAS/STING signaling in colon cancer cells. **(A, B)** MC38 cells were treated with 2 μM 5-FU **(A)** or 20 μM oxaliplatin (OXA) for the indicated time. Western blot analysis was performed to examine expression of STING, P-STING (S365), TBK1, P-TBK1 (S172), STAT1, P-STAT1 (Y701) as well as cGAS, ISG15 and γH2AX. **(C, D)** IFNβ and CXCL10 mRNA expression was determined by Q-PCR analysis in 5-FU- **(C)** or OXA- **(D)** treated MC38 cells. **(E)** CT26 cells were treated with 2 μM 5-FU or 20 μM oxaliplatin (OXA) for indicated time. Western blot analysis was performed to examine expression of STING, P-STING (S365), STAT1, P-STAT1 (Y701) as well as ISG15 and γH2AX. **(F, G)** IFNβ and CXCL10 mRNA expression was determined by Q-PCR analysis in 5-FU- **(F)** or OXA- **(G)** treated CT26 cells. *P<0.05, **P<0.01, ***P<0.001, ****P<0.0001.

5-FU and oxaliplatin can both cause DNA damage. After treatment of mouse colon cancer cells MC38 with 5-FU or oxaliplatin for different periods of time, the level of the well-known DNA damage marker γH2AX was significantly elevated (**Figures 1A, B**). Since DNA damage activates the cGAS/STING pathway (15–17), we determined whether 5-FU activates STING as well as downstream IFNβ/JAK/STAT signaling. We found that 5-FU increased phosphorylation of STING (P-STING), TBK1 (P-TBK1) and STAT1 (P-STAT1), consistent with the activation of cGAS/STING signaling (**Figures 1A, B**). In addition, 5-FU and oxaliplatin upregulated expression of IFNβ and CXCL10 in a time-dependent manner (**Figures 1C, D**). CXCL10 is one of the chemokines whose expression is regulated by IFNβ (34, 35) and is a key mediator that attracts antigen specific CD8^+^ T-cell migration (36). Expression of ISG15, one of the IFNβ-stimulated genes, was also increased in a time-dependent manner (**Figures 1A, B**).

To determine whether 5-FU and oxaliplatin have similar effect in other colon cancer cells, CT26 cells were treated with 5-FU or oxaliplatin for different periods of time. Like in MC38 cells, 5-FU and oxaliplatin increased levels of γH2AX, P-STING, P-TBK1 and P-STAT1 and induced expression of IFNβ, CXCL10 and ISG15 in CT26 cells (**Figures 1E–G**). Taken together, these results indicate that 5-FU and oxaliplatin activate the cGAS/STING pathway and increase downstream IFNβ/JAK/STAT signaling.

### 5-FU and oxaliplatin induced IFNβ/JAK/STAT signaling is dependent on cGAS/STING

**Figure 2:**
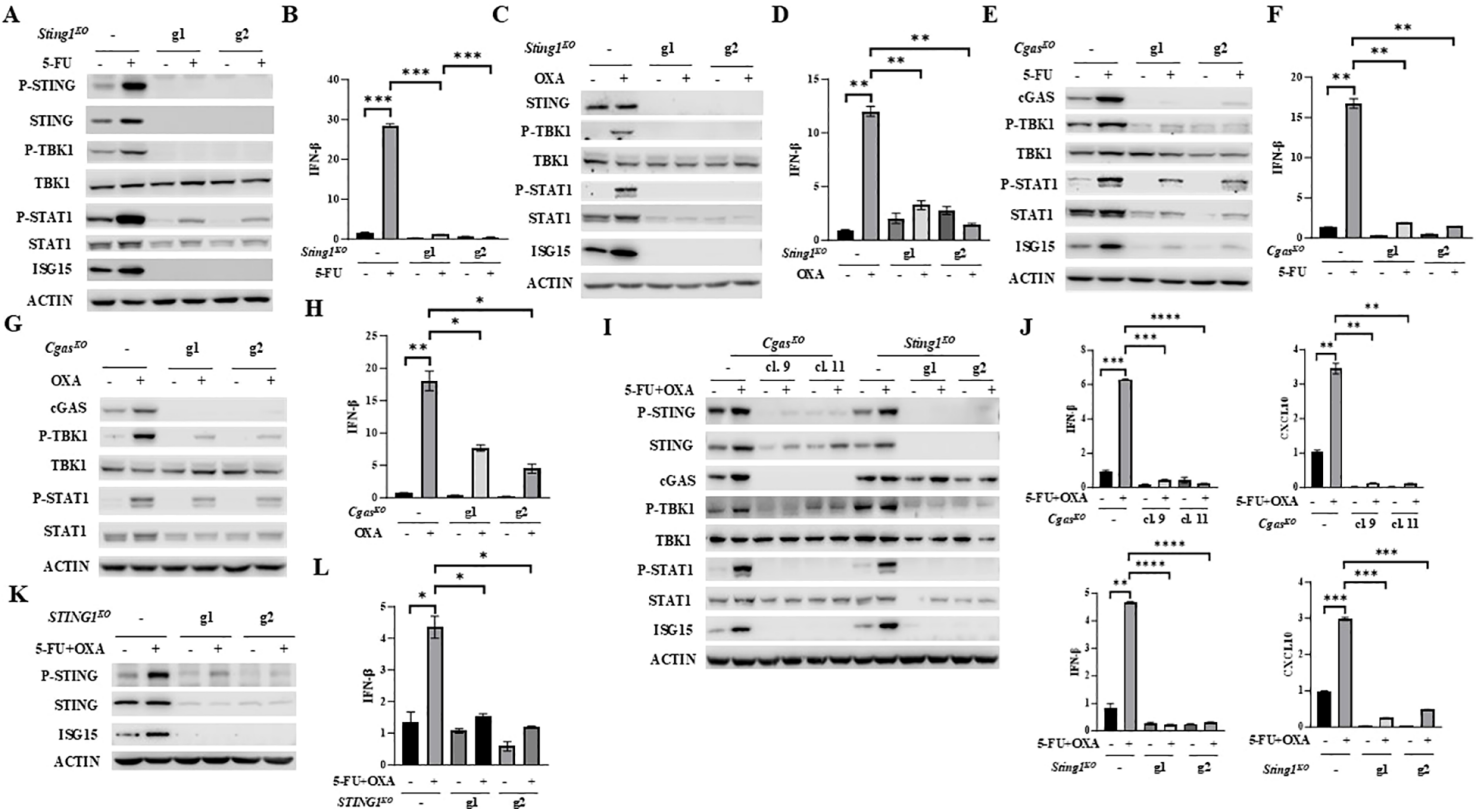
Depletion of STING or cGAS abolishes 5-FU/oxaliplatin mediated cGAS/STING activation in colon cancer cells. **(A-D)** *Sting1* knockout (*Sting1^KO^*) was performed by CRISPR/Cas9 in MC38 cells using two guide RNAs (g1 and g2). Cells were treated with 2 μM 5-FU or 20 μM oxaliplatin (OXA) for 24 hours. Western blot analysis was performed to examine expression of STING, P-STING (S365), TBK1, P-TBK1 (S172), STAT1, P-STAT1 (Y701) and ISG15 **(A, C)**. IFNβ expression was determined by Q-PCR analysis **(B, D)**. **(E-H)** *Cgas* knockout (*Cgas^KO^*) was performed by CRISPR/Cas9 in MC38 cells using two guide RNAs (g1 and g2). Cells were treated with 2 μM 5-FU or 20 μM oxaliplatin (OXA) for 24 hours. Western blot analysis was performed to examine expression of TBK1, P-TBK1 (S172), STAT1, P-STAT1 (Y701) and ISG15 **(E, G)**. IFNβ expression was determined by Q-PCR analysis **(F, H)**. **(I, J)** *Cgas* or *Sting1* knockout (*Cgas^KO^* or *Sting1^KO^*) was performed by CRISPR/Cas9 in CT26 using two guide RNAs. Single cell clones (#9 and #11) of *Cgas^KO^* cells were isolated. Other knockout cells were pools. Cells were treated with 2 μM 5-FU plus 10 μM oxaliplatin (OXA) for 24 hours. Western blot analysis was performed to examine expression of STING, P-STING (S365), TBK1, P-TBK1 (S172), STAT1, P-STAT1 (Y701), cGAS and ISG15 expression **(I)**. IFNβ and CXCL10 expression was determined by Q-PCR analysis **(J)**. **(K, L)** *STING1* was knocked out in HT29 cells by two gRNAs (g1 and g2). Cells were treated with 5 μM 5-FU plus 20 μM oxaliplatin (OXA) for 48 hours. Western blot analysis was performed to examine expression of STING, P-STING (S366) and ISG15 **(K)**. IFNβ expression was determined by Q-PCR analysis **(L)**. *P<0.05, **P<0.01, ***P<0.001, ****P<0.0001.

To establish whether 5-FU or oxaliplatin increases IFNβ/JAK/STAT signaling and induces the expression of IFNβ and CXCL10 through cGAS/STING activation, we substantially depleted STING or cGAS expression in MC38 cell pools by transfection with two independent guide RNAs (gRNAs: g1 and g2). Significantly diminished STING expression almost completely abrogated basal cellular levels as well as 5-FU- or oxaliplatin-induced P-STING, P-TBK1, P-STAT1, ISG15 and IFNβ expression (**Figures 2A–D**). An identical pattern of reduced basal and 5-FU- or oxaliplatin-induced cGAS/STING pathway activation was observed following depletion of cGAS (**Figures 2E–H**).

cGAS or STING was similarly reduced or eliminated in CT26 cells with independent gRNAs. Induction of P-STING, P-TBK1, P-STAT1, ISG15, IFNβ and CXCL10 expression by the combined treatment of 5-FU and oxaliplatin was diminished by depletion of cGAS or STING expression (**Figures 2I, J**). Furthermore, knockout of *STING1* in HT29 human colon cancer cells significantly attenuated 5-FU plus oxaliplatin-induced levels of P-STING and expression of ISG15 and IFNβ (**Figures 2K, L**). These results support the conclusion that 5-FU and oxaliplatin induced cytokine and chemokine signaling is dependent on cGAS and STING.

### 5-FU and oxaliplatin increase PD-L1 expression through cGAS/STING/IFNβ activation

**Figure 3:**
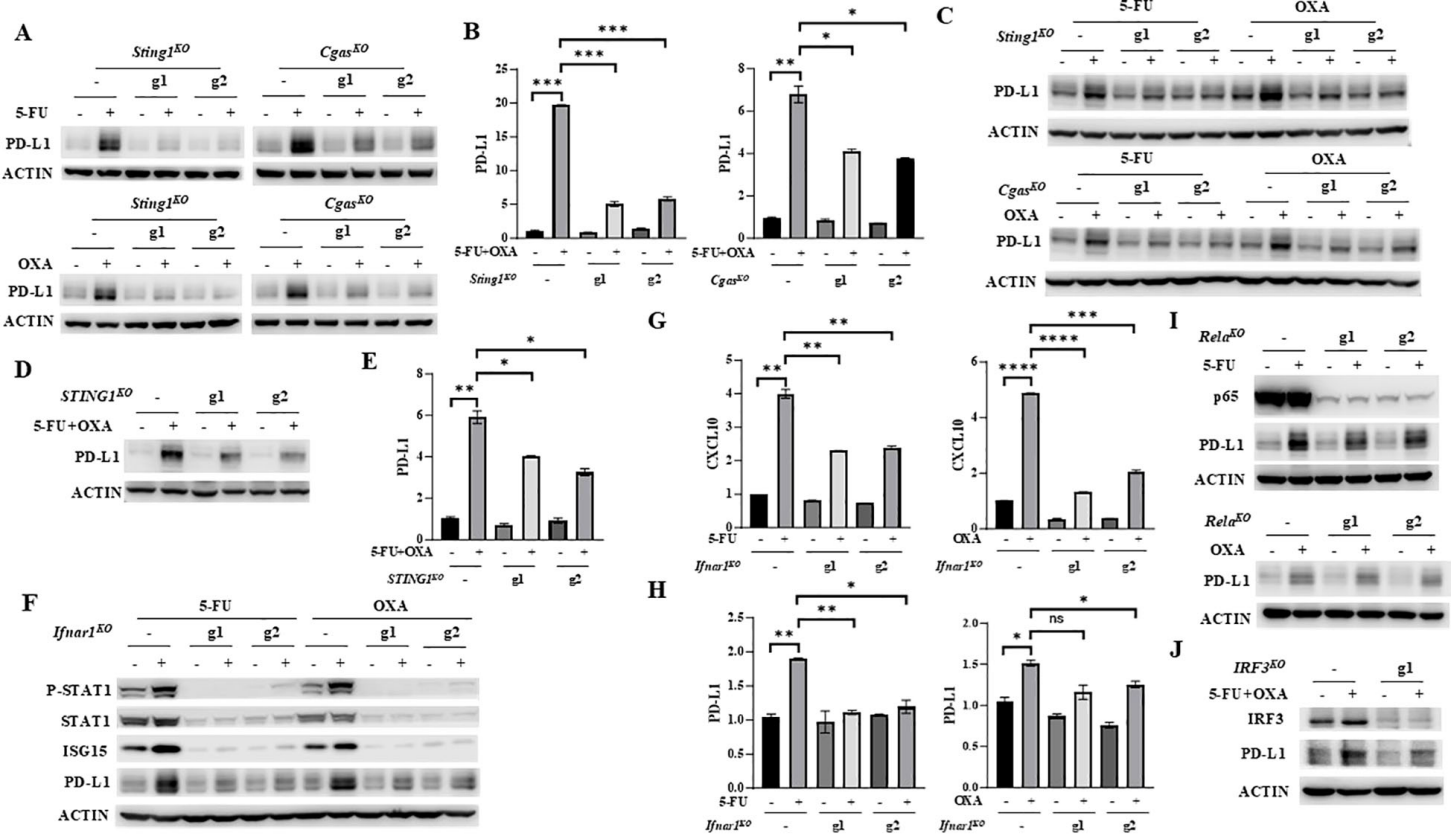
5-FU/oxaliplatin upregulates PD-L1 expression in a cGAS/STING/IFNβ-dependent manner in colon cancer cells. **(A, B)** *Sting1* or *Cgas* knockout (*Sting1^KO^* or *Cgas^KO^*) MC38 cells were treated with 2 μM 5-FU or 20 μM oxaliplatin (OXA) **(A)** or 2 μM 5-FU plus 10 μM oxaliplatin (OXA) **(B)** for 24 hours. Western blot **(A)** and Q-PCR **(B)** analysis were performed to examine PD-L1 expression. **(C)** *Sting1* or *Cgas* knockout (*Sting1^KO^* or *Cgas^KO^*) CT26 cells were treated with 2 μM 5-FU or 20 μM oxaliplatin (OXA) for 24 hours. Western blot analysis was performed to examine PD-L1 expression. **(D, E)**, *STING1* knockout (*STING1^KO^*) HT29 cells were treated with 5 μM 5-FU plus 20 μM oxaliplatin (OXA) for 48 hours. Western blot **(D)** and Q-PCR **(E)** analysis were performed to examine PD-L1 expression. **(F-H)** *Ifnar1* knockout (*Ifnar1^KO^*) MC38 cells were treated with 2 μM 5-FU or 20 μM oxaliplatin (OXA) for 24 hours. Western blot analysis was performed to examine expression of STAT1, P-STAT1 (Y701), ISG15 and PD-L1 expression **(F)**. Expression of CXCL10 **(G)** and PD-L1 **(H)** was determined by Q-PCR analysis. **(I)** *Rela* knockout (*Rela^KO^*) MC38 cells were treated with 2 μM 5-FU (upper panel) or 20 μM oxaliplatin (OXA, lower panel) for 24 hours. Western blot analysis was performed to examine expression of p65 and PD-L1. **(J)** *IRF3* knockout (*IRF3^KO^*) HT29 cells were treated with 5 μM 5-FU plus 20 μM oxaliplatin (OXA) for 48 hours. Western blot analysis was performed to examine expression of IRF3 and PD-L1. *P<0.05, **P<0.01, ***P<0.001, ****P<0.0001.

The observations that 5-FU and oxaliplatin activate the cGAS/STING signaling pathway suggest that the anti-tumor effect of FOLFOX may be partially attributed to cGAS/STING-mediated immune activation. However, cancer cells often escape immune regulation by inducing expression of PD-L1 (8, 9), which may be one of the mechanisms of chemoresistance in CRC. We next determined whether 5-FU and oxaliplatin affect PD-L1 expression. We found that 5-FU or oxaliplatin increased PD-L1 expression in MC38 cells at both mRNA and protein levels (**Figures 3A, B**). Moreover, depletion of STING or cGAS significantly attenuated 5-FU or oxaliplatin-induced PD-L1 expression (**Figures 3A, B**). Similar results were observed in CT26 cells (**Figure 3C**). In addition, depletion of STING expression in HT29 cells at least partially diminished PD-L1 upregulation at mRNA and protein levels by the combined treatment of 5-FU and oxaliplatin (**Figures 3D, E**).

IFNγ has been shown to regulate PD-L1 expression through the IFNγ-JAK/STAT axis (37). Whether IFNβ can regulate PD-L1 expression is unknown. IFNβ receptor forms a ternary complex, composed of its two subunits IFNAR1 and IFNAR2 that are both necessary for full IFNβ signaling (38, 39). To determine whether 5-FU and oxaliplatin regulate PD-L1 expression through IFNβ-mediated signaling, we depleted the IFNAR1 subunit of the IFNRI receptor using two independent gRNAs in MC38 cells. Eliminating IFNAR1 expression abolished both the basal levels and 5-FU- or oxaliplatin-induced upregulation of P-STAT1 and ISG15 (**Figure 3F**). The induction of CXCL10 expression by 5-FU or oxaliplatin was also attenuated in *Ifnar1* knockout cells (**Figure 3G**). These results indicated that depletion of IFNAR1 abolishes IFNβ downstream signaling. Moreover, abrogation of IFNβ/IFNAR1/STAT signaling eliminated upregulation of PD-L1 protein and mRNA expression by 5-FU or oxaliplatin (**Figures 3F, H**). These results indicate that treatment by 5-FU or oxaliplatin enhances PD-L1 expression through the cGAS/STING/IFNβ/IFNAR/JAK/STAT signaling axis.

IRF3 and NF-κB are two transcription factors that can be activated by cGAS/STING signaling to induce IFNβ expression (13, 14). To further dissect their contribution to the regulation of PD-L1 expression, p65-encoding gene, *Rela* was knocked out by two independent guide RNAs (g1 and g2) using CRISPR/Cas9 in MC38 cells. Depletion of p65 did not affect upregulation of PD-L1 by 5-FU and oxaliplatin (**Figure 3I**), indicating that NF-κB does not contribute to PD-L1 regulation. We also depleted IRF3 expression in HT29 cells using CRISPR/Cas9. The results showed that reduction of IRF3 attenuated upregulation of PD-L1 by 5-FU and oxaliplatin (**Figure 3J**). Taken together, these studies indicate that IRF3, but not NF-κB, contributes to PD-L1 upregulation by 5-FU and oxaliplatin.

**Figure 4:**
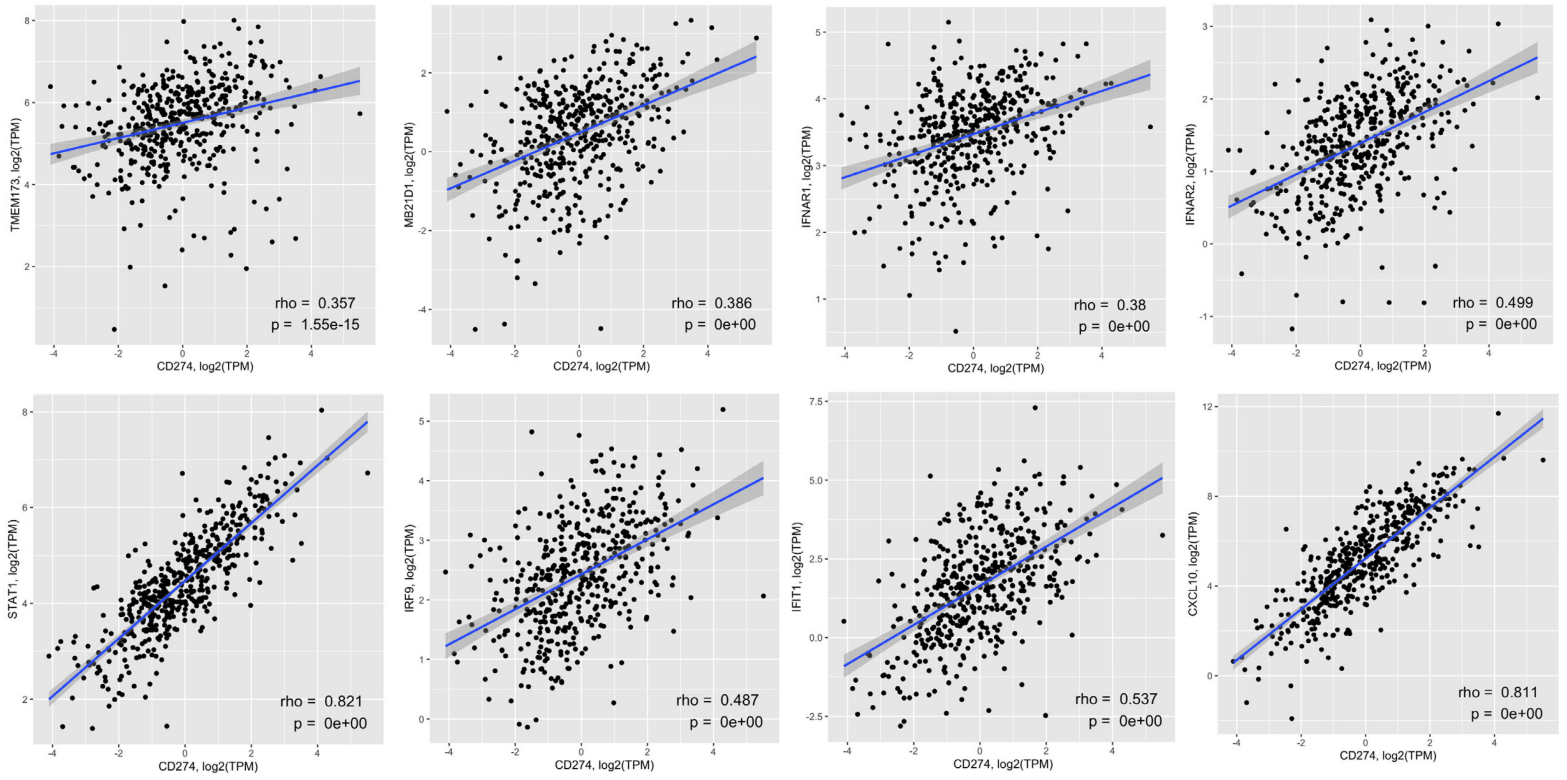
PD-L1 expression is positively associated with expression of cGAS/STING/IFNβ signaling components in colon cancer patient specimens. Correlation between expression of PD-L1 (*CD274*) and STING (*TMEM173*), cGAS (*MB21D1*), IFNAR1, IFNAR2, STAT1, IRF9, IFIT1 or CXCL10 in colon cancer patient samples from TCGA. Correlation of PD-L1 with other genes was calculated with Spearman’s correlation and p values.

To examine the clinical relevance of our findings, we determined the relationship between PD-L1 expression and cGAS/STING signaling in human colon cancer by mining RNA-seq data from TCGA colon cancer dataset (TCGA-COAD). Expression of PD-L1 (CD274) was positively correlated with expression of cGAS/STING/IFNβ signaling components, STING (TMEM173), cGAS (MB21D1), IFNAR1, IFNAR2, STAT1 and IRF9, and IFNβ-regulated genes and cytokines, IFIT1 and CXCL10 (**Figure 4**). These results suggest that cGAS/STING activation in colon cancer patients likely results in elevated PD-L1 expression and that upregulation of PD-L1 by 5-FU and oxaliplatin may be one of the mechanisms of chemoresistance in colon cancer treatment.

### Combination of 5-FU/oxaliplatin and an anti-PD-1 antibody effectively inhibits tumor growth in syngeneic immune competent mice

**Figure 5:**
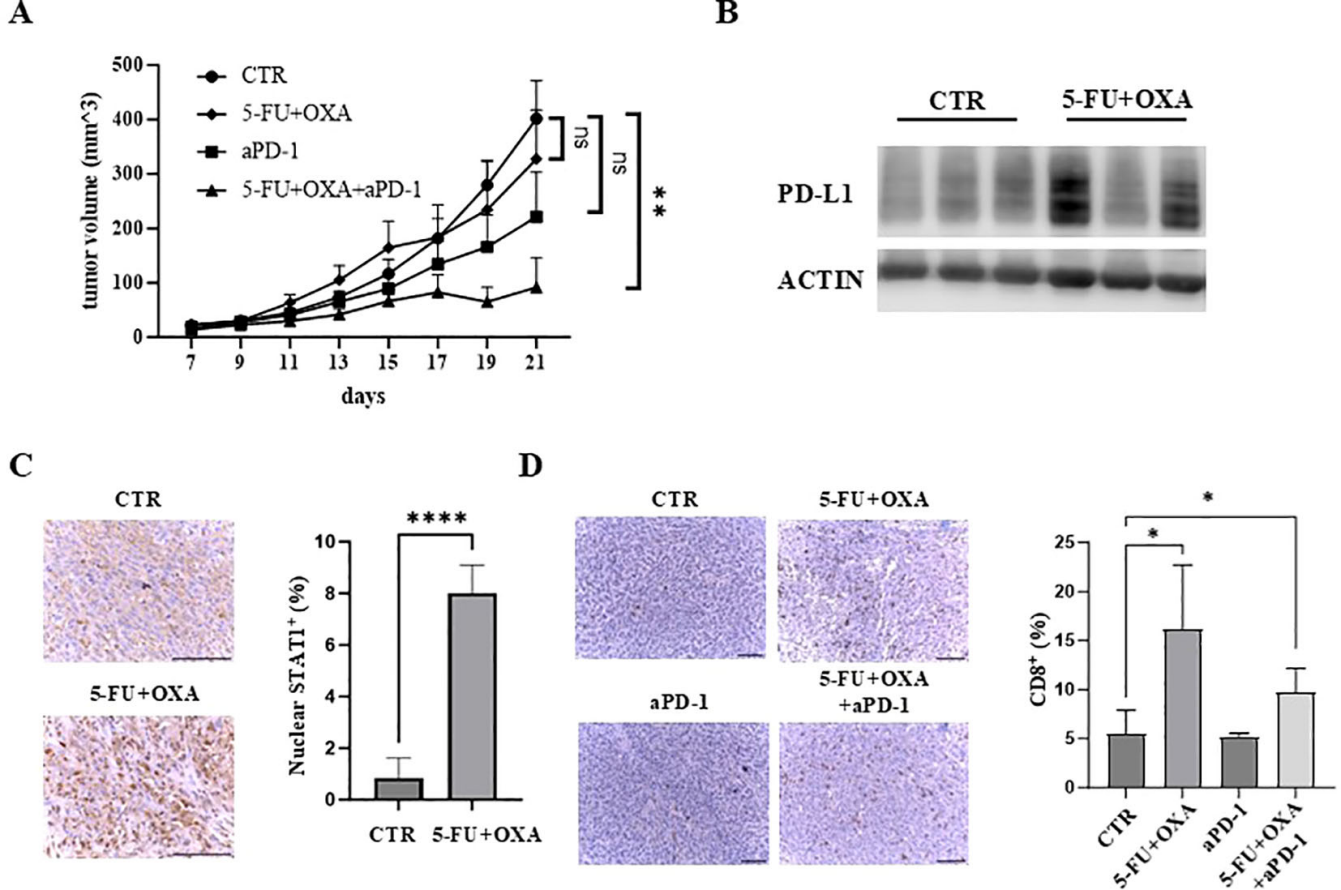
The combination of 5-FU/oxaliplatin and anti-PD-1 treatment efficiently inhibit tumor growth *in vivo*. **(A)** Tumor growth curves of MC38 cells treated with saline/a control IgG (CTR), 5-FU (25 mg/kg) plus oxaliplatin (OXA, 2.5 mg/kg) (5-FU+OXA), an anti-PD-1 antibody (200 μg) (aPD-1) or 5-FU (25 mg/kg)/oxaliplatin (OXA, 2.5 mg/kg) plus an anti-PD-1 antibody (200 μg) (5-FU+OXA+aPD-1) are shown. N = 5-10. **(B)** Western blot analysis was performed to examine PD-L1 expression in vehicle- (CTR) or 5-FU/oxaliplatin-treated (5-FU+OXA) tumors. **(C)** Representative images of IHC staining of STAT1 in control (CTR) and 5-FU/oxaliplatin (5-FU+OXA) treated tumors. Scale bars: 100 μm (left). Quantification of percentage of nuclear STAT1-positive cells is shown in the right panel. **(D)** Representative images of IHC staining of CD8 in tumors of each group. Scale bars: 100 μm (left). Quantification of percentage of CD8-positive cells is shown in the right panel. *P<0.05, **P<0.01, ****P<0.0001.

Given that 5-FU/oxaliplatin activates cGAS/STING signaling and elevates PD-L1 expression, we hypothesized that the combination of 5-FU/oxaliplatin and anti-PD-1 treatment would effectively inhibit tumor growth in immune competent mice. To test this premise, MC38 cells were injected subcutaneously into wild type C57BL*/*6 mice. These mice were then randomly divided into four groups and treated with 5-FU/oxaliplatin, anti-PD-1, 5-FU/oxaliplatin plus anti-PD-1 or vehicle control, respectively. Tumor growth was monitored, and tumor growth curves were generated. We found that treatment with 5-FU/oxaliplatin/anti-PD-1 combination significantly reduced tumor growth as compared to the control while 5-FU/oxaliplatin or anti-PD-1 treatment alone did not (**Figure 5A**). We determined PD-L1 expression in 5-FU/oxaliplatin-treated tumors and found that most 5-FU/oxaliplatin-treated tumors expressed much higher PD-L1 than vehicle-treated control tumors (**Figure 5B**). These results are consistent with the hypothesis that inhibition of the PD-1/PD-L1 immune check point mitigates 5-FU/oxaliplatin-induced upregulation of PD-L1, ultimately enhancing chemotherapy efficacy in this mouse model of CRC.

Immunohistochemical (IHC) staining of STAT1, a downstream effector of cGAS/STING signaling, showed that nuclear STAT1 was increased almost 8-fold following 5-FU/oxaliplatin treatment (**Figure 5C**). Enhanced nuclear localization of STAT1 is a significant indicator of cGAS/STING activation resulting in downstream cytokine and chemokine expression (13, 14, 40). Furthermore, we examined the extent of tumor infiltrating CD8^+^ T cells, a central player in anti-tumor immunity. IHC staining of tumor sections with an anti-CD8 antibody showed a marked increase of CD8^+^ T cells in 5-FU/oxaliplatin or 5-FU/oxaliplatin/anti-PD-1-treated tumors as compared to control or anti-PD-1-treated tumors (**Figure 5D**, left panel). Quantification of IHC stained cells indicated an approximate 3.1 or 1.9-fold increase in CD8^+^ T cells following 5-FU/oxaliplatin or 5-FU/oxaliplatin/anti-PD-1 treatment, respectively (**Figure 5D**, right panel). These results indicate that 5-FU/oxaliplatin treatment activates cGAS/STING signaling, leading to the recruitment of CD8^+^ T cells to the tumors, enhancing the efficacy of anti-PD-1 treatment.

**Figure 6:**
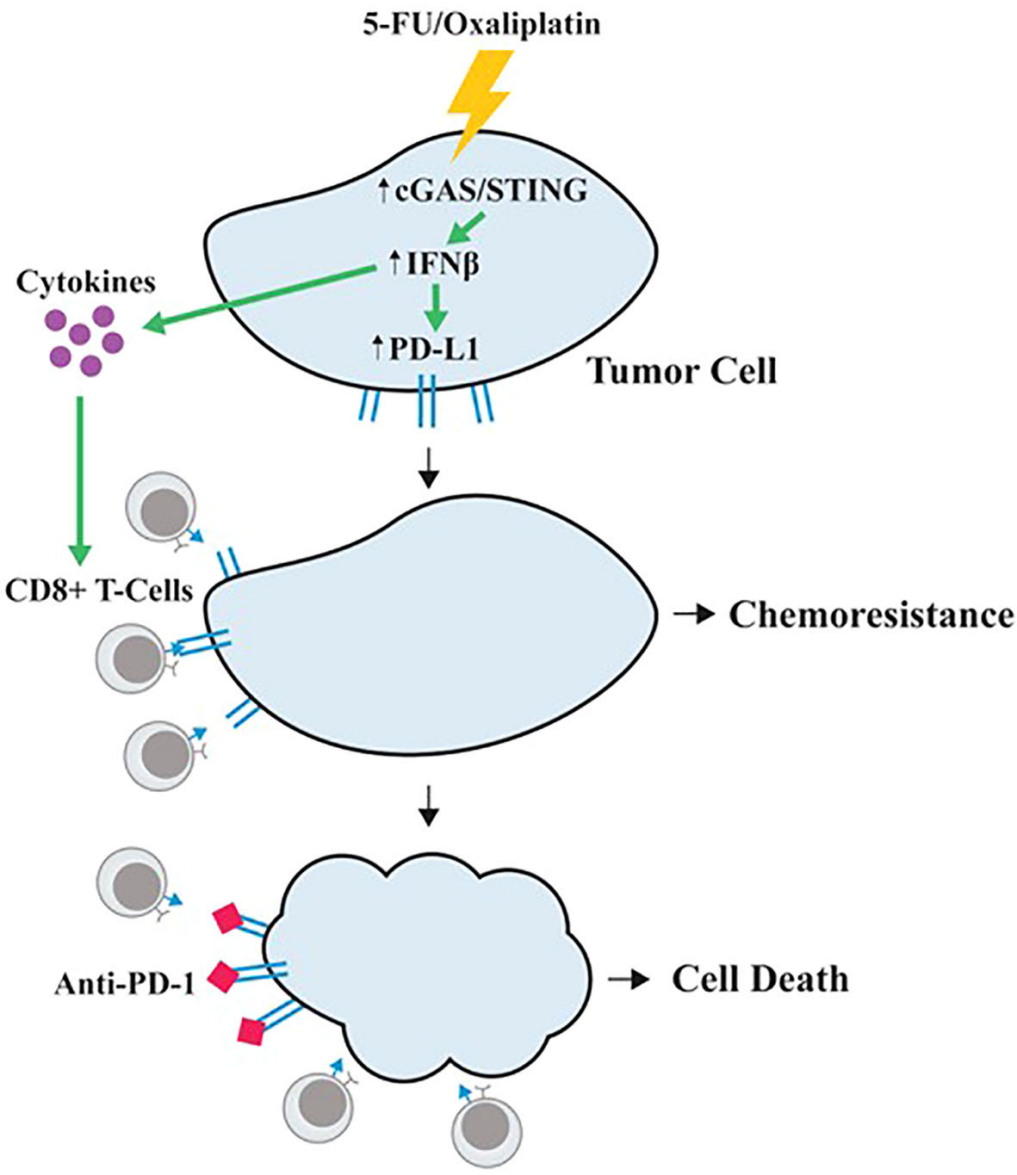
A proposed model of 5-FU/oxaliplatin function in cancer treatment. 5-FU/oxaliplatin activates the cGAS/STING innate immune response. On one hand, it recruits CD8^+^ T-cells to the TME, facilitating the elimination of tumor cells. On the other hand, it upregulates PD-L1 expression on tumor cells to evade immune surveillance, leading to chemoresistance. In contrast, combination of 5-FU/oxaliplatin with anti-PD-1 mitigates PD-L1-mediated immune escape, resulting in tumor cell death.

Taken together, our studies suggest a unique model of 5-FU/oxaliplatin function in cancer treatment (**Figure 6**). In this model, 5-FU/oxaliplatin activates the cGAS/STING innate immune response. On one hand, it recruits CD8^+^ T-cells to the TME, facilitating the elimination of tumor cells. On the other hand, it upregulates PD-L1 expression on tumor cells to evade immune surveillance, leading to chemoresistance. In contrast, the combination of 5-FU/oxaliplatin with anti-PD-1 mitigates PD-L1-mediated immune escape resulting in tumor cell death. Thus, the combined use of FOLFOX with anti-PD-1/PD-L1 may represent an effective therapeutic approach in CRC treatment.

## Discussion

Tumors almost universally escape immune regulation early in their development (41–43). Increased expression of PD-1/PD-L1 is one of many mechanisms exploited by tumors to escape anti-tumor immune surveillance (44–46). Inhibitors targeting PD-1/PD-L1 immune checkpoint has improved patient survival including CRC. However, 60-80% of patients do not respond to immune checkpoint treatment (3–5). Lack of TILs in CRC has been linked to immune checkpoint resistance (20–22). Therefore, at least two requirements must be necessarily met to have an efficient cancer therapy: 1) recruitment of TILs and 2) blockade of adaptive immune inhibitory signals such as the PD-1/PD-L1 checkpoint. In this study, we show that the main chemotherapeutic agents for first line treatment of CRC, 5-FU and oxaliplatin, activate cGAS/STING signaling, which facilitates TILs (19). Conversely, 5-FU and oxaliplatin treatment increases PD-L1 expression, which suppresses cytotoxic T cell function (6–8). Furthermore, combination of FOLFOX with an anti-PD-1 antibody significantly suppressed tumor growth in immune competent mice. These results suggest that cGAS/STING activation may contribute to FOLFOX anti-tumor activity and that induction of PD-L1 expression may be one of the mechanisms of chemoresistance in CRC. Taken as a whole, these observations suggest that the combined use of FOLFOX with anti-PD-1/PD-L1 may meet both requirements and represent an effective therapeutic approach.

It has been shown that IFNγ, derived from TILs, induces PD-L1 expression on tumor cells and/or in TME (37, 47). We show here that 5-FU and oxaliplatin upregulate PD-L1 expression in colon cancer cells. Depleting cGAS, STING or IFNAR1 expression largely abrogates PD-L1 induction, indicating that 5-FU and oxaliplatin regulate PD-L1 expression at least partly through the activation of cGAS/STING/IFNβ signaling. These observations suggest that PD-L1 may be regulated by IFNβ. Thus, in addition to TIL-derived IFNγ, tumor cell-derived IFNβ appears to be another mechanism for controlling PD-L1 expression. Perhaps more importantly, our studies suggest that activation of tumor cell intrinsic cGAS/STING signaling is likely to be a double-edged sword. On one hand it increases tumor infiltration of CD8^+^ T cells to facilitate anti-tumor immunity, and on the other hand induces PD-L1 expression to suppress T cell function. Hence, our studies also provide a rationale to combine STING agonists with anti-PD-1/PD-L1 in cancer treatment instead of using either treatment alone.

cGAS/STING activation is essential for efficient cancer therapy including chemotherapy and anti-PD-1/PD-L1 therapies (18, 19). As the cGAS/STING pathway is frequently suppressed or defective in a variety of cancers (18, 19, 23), the anti-tumor activity of FOLFOX, anti-PD-1/PD-L1 or their combination may show reduced efficacy in patients with defective cGAS/STING signaling. Identification of mechanisms by which cGAS/STING signaling is suppressed in CRC and find ways to restore or enhance cGAS/STING expression and activation would improve efficacy of FOLFOX, anti-PD-1/PD-L1 or their combination and overcome resistance. Methylation of the *STING1* or *CGAS* promoter has been reported in different cancers and inhibitors of DNA methyltransferase restored STING signaling (18, 48, 49). We have shown that SIX4, a transcription factor, upregulates STING expression and enhances anti-PD-1 efficacy in colon cacner cells (26). Thus, targeting mechanisms of cGAS/STING suppression to reactivate its signaling might be helpful to increase effectiveness of chemo- and/or immuno-therapies.

Although both 5-FU and oxaliplatin induce DNA damage, the underlying mechanisms are different. 5-FU mainly inhibits thymidylate synthase and blocks the synthesis of dTMP required for DNA replication (27) whereas oxaliplatin forms intra-strand DNA adducts and disrupts DNA replication and transcription (28). Nevertheless, they both activate cGAS/STING signaling efficiently and increase PD-L1 expression. Analysis of TCGA colon cancer dataset shows a positive correlation between expression of PD-L1 and components of the cGAS/STING pathway, providing clinical support for cGAS/STING signaling in upregulating PD-L1 expression. Tumor studies in syngeneic immune competent mice show that the combination of 5-FU/oxaliplatin and anti-PD-1 significantly reduced tumor growth of colon cancer cells in immune competent mice as compared to 5-FU/oxaliplatin treatment alone. Taken together, our studies identify a unique pathway leading to chemoresistance and provide a rationale to combine FOLFOX with anti-PD-1/PD-L1 as an effective CRC treatment.

## Data Availability

The original contributions presented in the study are included in the article/Supplementary Material. Further inquiries can be directed to the corresponding author.
